# Supplementary material for: Preventive and Therapeutic Effects of Lactiplantibacillus plantarum HD02 and MD159 through Mast Cell Degranulation Inhibition in Mouse Models of Atopic Dermatitis
Source: Nutrients. 2024 Sep 6;16(17):3021. doi: 10.3390/nu16173021 (PMC11396792; doi:10.3390/nu16173021)
Supplement: Supplementary file 1 [file nutrients-16-03021-s001.zip › nutrients-3133593-supplementary.pdf]

Supplementary Materials

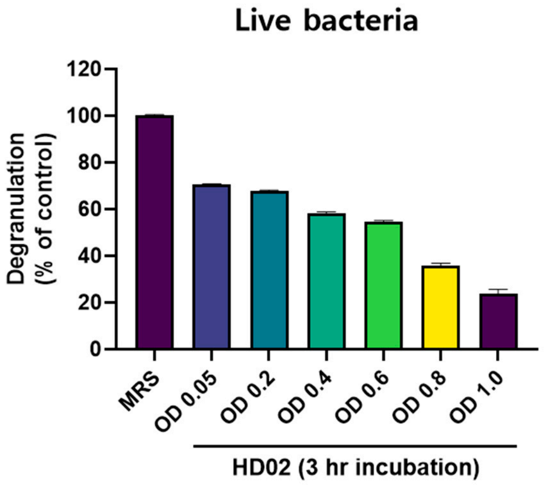

Supplementary Figure S1. Dose-dependent inhibition of mast cell degranulation by *L. plantarum* HD02. The pellet of *L. plantarum* HD02 obtained immediately after culture was washed with PBS and then resuspended in S-buffer to prepare various doses of *L. plantarum* HD02 by adjusting the OD_{600nm} values were 0.05, 0.2, 0.4, 0.6, 0.8, and 1.0, respectively. Then, each of these various doses of *L. plantarum* HD02 was treated to mast cells for 3 hours to investigate its inhibitory ability on mast cell degranulation. Data are represented as mean±SD.

Strain	Taxon	Viable cell count (CFU/mL)
MK07	*Lactobacillus acidophilus*	1.78×10^8
MD41	*Lactobacillus brevis*	6.70×10^7
MD167	*Lactobacillus brevis*	5.00×10^8
HD03	*Lactobacillus fermentum*	4.90×10^8
MD8	*Lactobacillus fermentum*	6.30×10^7
MD58	*Lactobacillus fermentum*	3.10×10^7
MD105	*Lactobacillus fermentum*	6.00×10^8
MD154	*Lactobacillus fermentum*	5.50×10^8
MK03	*Lactobacillus gasseri*	9.70×10^7
MD20a	*Lactobacillus paracasei*	6.20×10^8
MD57	*Lactobacillus paracasei*	1.82×10^8
MD120	*Lactobacillus paracasei*	3.10×10^8
HD02	*Lactobacillus plantarum*	3.50×10^8
MD15	*Lactobacillus plantarum*	6.10×10^7
MD152	*Lactobacillus plantarum*	3.60×10^8
MD153	*Lactobacillus plantarum*	1.24×10^7
MD157	*Lactobacillus plantarum*	4.20×10^7
MD159	*Lactobacillus plantarum*	1.44×10^8
MD161	*Lactobacillus plantarum*	1.00×10^8
MD3	*Lactobacillus rhamnosus*	4.30×10^8
MD29	*Lactobacillus rhamnosus*	4.30×10^8
MD33	*Lactobacillus rhamnosus*	5.90×10^8
MD44	*Lactobacillus rhamnosus*	4.40×10^8
MD49	*Lactobacillus rhamnosus*	4.00×10^8
MD150	*Lactobacillus rhamnosus*	3.50×10^8

Supplementary Table S1. Viable cell count of each bacterial strain when the OD_{600nm} value is 0.8.
